# State-Dependent Transcranial Magnetic Stimulation Synchronized with Electroencephalography: Mechanisms, Applications, and Future Directions

**DOI:** 10.3390/brainsci15070731

**Published:** 2025-07-08

**Authors:** He Chen, Tao Liu, Yinglu Song, Zhaohuan Ding, Xiaoli Li

**Affiliations:** 1 School of Automation Science and Engineering, South China University of Technology, Guangzhou 510641, China; chenhe@scut.edu.cn (H.C.); auliutao@mail.scut.edu.cn (T.L.); ausongyinglu@mail.scut.edu.cn (Y.S.); xiaolili@scut.edu.cn (X.L.); 2 Shien-Ming Wu School of Intelligent Engineering, South China University of Technology, Guangzhou 510640, China

**Keywords:** transcranial magnetic stimulation, TMS-EEG, state-dependent, closed-loop

## Abstract

Transcranial magnetic stimulation combined with electroencephalography (TMS-EEG) has emerged as a transformative tool for probing cortical dynamics with millisecond precision. This review examines the state-dependent nature of TMS-EEG, a critical yet underexplored dimension influencing measurement reliability and clinical applicability. By integrating TMS’s neuromodulatory capacity with EEG’s temporal resolution, this synergy enables real-time analysis of brain network dynamics under varying neural states. We delineate foundational mechanisms of TMS-evoked potentials (TEPs), discuss challenges posed by temporal and inter-individual variability, and evaluate advanced paradigms such as closed-loop and task-embedded TMS-EEG. The former leverages real-time EEG feedback to synchronize stimulation with oscillatory phases, while the latter aligns TMS pulses with task-specific cognitive phases to map transient network activations. Current limitations—including hardware constraints, signal artifacts, and inconsistent preprocessing pipelines—are critically analyzed. Future directions emphasize adaptive algorithms for neural state prediction, phase-specific stimulation protocols, and standardized methodologies to enhance reproducibility. By bridging mechanistic insights with personalized neuromodulation strategies, state-dependent TMS-EEG holds promise for advancing both basic neuroscience and precision medicine, particularly in psychiatric and neurological disorders characterized by dynamic neural dysregulation.

## 1. Introduction

Since Barker and his research team first applied high-intensity alternating magnetic fields to stimulate the human cerebral cortex in 1985 [1], transcranial magnetic stimulation (TMS) has gradually been utilized in clinical treatments for various neurological disorders as a safe, non-invasive brain modulation technique [2,3]. TMS-assisted rehabilitation has been implemented in conditions including schizophrenia [4], Parkinson’s syndrome [5], Alzheimer’s disease [6], amyotrophic lateral sclerosis [7], neuropathic pain [8], major depression [9], and other related disorders [10]. The synchronous combination of TMS with electroencephalography (EEG) provides a novel technology for detecting brain neurofunctional states. Variations in neural states at the moment of TMS stimulation can lead to differences in TMS-EEG measurements. The state-dependent problem of TMS-EEG aims to investigate how the relative relationship between the timing of TMS stimulation and neural activity states affects TMS-EEG measurement outcomes, as well as to propose or develop new technologies for optimizing existing TMS-EEG protocols.

This review synthesizes current advancements in state-dependent TMS-EEG research. First, we elucidate the biophysical principles underlying TMS-EEG and the neurophysiological origins of TMS-evoked potentials (TEPs). Next, we analyze how neural states modulate TMS effects, emphasizing closed-loop systems that synchronize stimulation with EEG-derived biomarkers and task-embedded paradigms that probe cognition-dependent network dynamics. We further discuss technical barriers, including artifact mitigation and real-time signal processing challenges, and propose strategies to enhance reproducibility through adaptive algorithms and standardized pipelines. By contextualizing these innovations within clinical and cognitive frameworks, this work aims to establish a roadmap for translating state-dependent TMS-EEG from experimental tools to reliable biomarkers and targeted therapies.

## 2. TMS and TMS-EEG

### 2.1. TMS

For each transmitted TMS pulse, the TMS device generates high-slope, high-intensity current pulses in the stimulation coil, producing a proportional induced magnetic field around the coil. As a magnetic field stimulation modulation method [11], TMS operates based on electromagnetic induction principles to activate intracranial neurons. The induced magnetic field penetrates the scalp, skull, and brain tissue, generating secondary induced currents in the cortical gyri. These currents alter the membrane potentials of neurons in the target region, leading to activation or inhibition of related brain areas. The most direct manifestation of TMS effects is the generation of motor evoked potentials (MEPs). When a suprathreshold TMS pulse is applied to the primary motor cortex, transient contraction of corresponding peripheral muscles occurs, and MEP signals can be recorded. MEP amplitude increases with TMS intensity, and the motor threshold is defined as the minimum TMS output required to elicit MEPs. Specifically, this threshold is met when resting-state MEP amplitudes exceed 50 μV in at least 5 out of 10 trials or task-state MEP amplitudes (with 20% contraction of hand target muscles) exceed 200 μV [12].

Based on stimulation patterns, TMS can be classified into single-pulse TMS (sTMS) [13], paired-pulse TMS (pTMS) [14], and repetitive TMS (rTMS) [15]. sTMS and pTMS are often combined with other neuroimaging tools [16,17] as diagnostic methods, while rTMS is primarily used for neuromodulation [18]. By combining sTMS with MEPs, researchers assess corticospinal excitability changes through MEP amplitude [19] or calculate metrics like central motor conduction time (CMCT) to detect pathological changes in neural pathways [20]. pTMS involves two consecutive pulses, where the first pulse acts as a conditioning stimulus (CS) to modulate the effect of the second test stimulus (TS). The influence of CS on TS varies depending on the inter-pulse interval [21]. By integrating pTMS with MEPs, researchers investigate intracortical and intercortical facilitation/inhibition mechanisms through metrics such as short-interval intracortical inhibition (SICI), long-interval intracortical inhibition (LICI), short-interval intracortical facilitation (SICF), contralateral silent period (SP), and ipsilateral silent period (iSP) [10]. TMS can also be combined with peripheral nerve stimulation (e.g., median nerve stimulation, MNS) to form paired associative stimulation (PAS) [22], exploring corticospinal-peripheral interactions [23].

rTMS is categorized into high-frequency rTMS (>1 Hz) and low-frequency rTMS (≤1 Hz), both widely used in neurological rehabilitation and cognitive enhancement. Previous studies suggest that high-frequency rTMS increases cortical excitability [24], while low-frequency rTMS reduces it [25]. By fixing the TMS pulse pattern to mimic the brain’s theta rhythm, a novel paradigm called theta-burst stimulation (TBS) [26] was developed. TBS delivers three 50 Hz pulses per burst at 5 bursts per second. Compared to rTMS, TBS achieves equivalent therapeutic effects with significantly shorter treatment durations for similar indications [27].

The stimulation coil is a critical component of the TMS device, serving as the magnetic field emitter. Coil design directly impacts electromagnetic field distribution, determining stimulation focus, depth, and intensity [28]. The most common coil types are circular and figure-of-eight coils [29]. The figure-of-eight coil consists of two adjacent circular coils with opposing current directions, generating peak electric fields at the intersection. Cohen et al. demonstrated its superior spatial focus compared to circular coils in spherical head models [30]. TMS combined with neuronavigation systems reduces variability in stimulation effects caused by coil positioning [31]. Navigated TMS uses anatomical or fMRI structural data with optical tracking to maintain millimeter-level spatial precision [32]. Julkunen et al. demonstrated that navigated TMS produces more stable inter-trial MEP recordings compared to non-navigated protocols [33].

### 2.2. TMS-EEG

As early as 1989, Cracco et al. first demonstrated the feasibility of combining TMS with EEG by stimulating the frontal cortex and recording contralateral EEG responses [34]. In this study, TMS evoked stable, positive-polarity EEG components in contralateral regions with onset latencies of 8.8–12.2 ms, durations of 7–15 ms, and amplitudes up to 20 μV, marking the birth of TMS-EEG. A typical TMS-EEG experimental setup is that participants wear TMS-compatible EEG caps, receive over 100 TMS pulses at intervals ≥3 s, and record EEG responses. High-resolution experiments may incorporate navigation systems with fMRI structural data to ensure precise targeting [35].

When TMS pulses are applied to the cortex, time-locked depolarization occurs in targeted neurons, followed by trans-synaptic activation of local and distal networks. Scalp electrodes record characteristic positive/negative EEG deflections, termed TEPs [36]. While the exact origin of TEPs remains debated, they are widely attributed to spatiotemporal summation of excitatory and inhibitory postsynaptic potentials from pyramidal and interneuron activity [37]. Pharmacological studies using randomized, placebo-controlled, crossover, and double-blind designs help elucidate TEP mechanisms. For example, recording TEPs before and after administering GABAergic drugs reveals the critical roles of GABAA and GABAB receptors in shaping TEP components [38]. TEPs reflect cortical reactivity, excitability, and coherence, with amplitude and latency changes indicating altered neural dynamics [39,40]. Linking TEPs to specific disorders or behavioral tasks offers new insights into brain-behavior relationships.

Beyond time-domain TEP analysis, TMS-EEG data can be explored through time-frequency analysis [41], connectivity [42], and brain network metrics [43,44], providing multidimensional insights into neural states under TMS perturbation. TMS-induced temporal response patterns help define causal connectivity across regions, revealing cortical network excitability, oscillatory tuning, and connectivity [45]. For instance, analyzing single-trial TMS-EEG data may show that activity in region A precedes region B, suggesting excitatory or inhibitory interactions between them. TMS impacts neural networks through two approaches: virtual lesion and probe. The virtual lesion method disrupts normal activity during task execution via single or rapid pulse trains, involving inhibitory neuron activation, neuronal noise injection, or task-irrelevant neuron recruitment [46]. The probe method perturbs resting or task-active regions while recording EEG responses to map network propagation [47], elucidating neural pathway dynamics [48].

Compared to early MEP-based approaches, TMS-EEG significantly expands the scope of neurophysiological insights. Combining TMS and EEG establishes non-invasive, safe methods to assess structural states, neural excitability, and cortical dynamics. TMS-EEG enables millisecond-scale investigations of cortico-cortical interactions, plasticity, and excitation-inhibition balance [49]. It has emerged as a powerful clinical tool for evaluating psychiatric and neurological disorders (e.g., schizophrenia, bipolar disorder, vegetative states) [50], consciousness levels (e.g., anesthesia, sleep, vigilance) [51], and treatment-induced neural changes. A critical requirement for TMS-EEG biomarkers is high reliability and reproducibility [52]. Reproducibility entails consistency across: (1) Different TMS devices under identical conditions; (2) different participants within the same cohort; and (3) repeated measurements in the same participant over time.

Despite robust characterization in healthy populations, TMS-EEG variability remains a challenge, stemming from methodological differences (e.g., current direction and pulse waveform) [53], neuropsychological states, physiological factors (age, sex, medication, alcohol use), recording timing, sleep status, and menstrual cycle phases [54]. Strategies to reduce variability include restricting participant demographics (age, sex), prohibiting psychoactive substances (alcohol, caffeine), standardizing preprocessing/analysis pipelines, and using high-focus coils and navigation systems [55]; in addition, using source-space TEPs to account for individual differences in cortical anatomy is more reliable than conventional TEPs [56]. Recent studies propose improving reproducibility by ensuring consistent neural states at TMS delivery, thereby reducing neural variability [57,58,59].

## 3. State-Dependent TMS-EEG Technology

A widely accepted view is that the impact of TMS pulses on the nervous system depends not only on TMS stimulation parameters but also on the brain state of the targeted region at the moment of TMS delivery [60]. By using real-time physiological signal feedback to control TMS timing or administering TMS during task-specific experiments, neural activity states can be “locked” to acquire more meaningful TMS-EEG data. The key to implementing state-dependent TMS-EEG lies in clarifying the characteristics of TMS-EEG under various stimulation timings and determining how to select TMS timing to achieve desired experimental outcomes.

### 3.1. Closed-Loop TMS-EEG and Task-Embedded TMS-EEG

**Figure 1:**
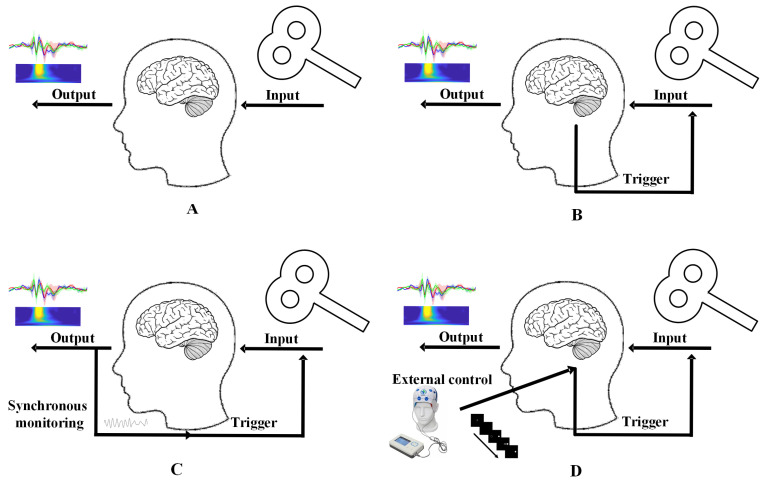
Open-loop vs. closed-loop TMS technologies. (**A**) Open-loop TMS, (**B**) closed-loop TMS, (**C**) direct closed-loop TMS, (**D**) induced closed-loop TMS.

Despite significant advancements in TMS technology over the past four decades—providing a safe and well-tolerated treatment for neurological and psychiatric disorders—TMS-EEG studies often exhibit high variability. Both therapeutic efficacy and screening reliability are hindered by poorly understood intra- and inter-individual variability. Efforts to personalize TMS protocols for specific disorders aim to improve treatment outcomes. In neuromodulation research, the brain is often treated as a “black box” where stimulation represents the input and neural activity represents the output. Neuromodulation can be categorized into two approaches:

(1) Open-loop modulation applies predefined stimulation protocols without real-time feedback from brain states (Figure 1A). Traditional TMS protocols fall into this category.
(2) Closed-loop modulation adjusts stimulation based on instantaneous neural activity, allowing brain “outputs” to influence inputs (Figure 1B) [61]. Closed-loop systems standardize initial states and investigate how specific brain states modulate stimulation effects. TMS protocols guided by real-time neural states are termed closed-loop TMS.

For closed-loop TMS, the most direct approach involves recording spontaneous neural signals (e.g., EEG) to guide TMS delivery in real time (Figure 1C). Integrating TMS with EEG enables protocols controlled by EEG-derived brain states, offering a pathway to stable, personalized neuromodulation. Challenges include real-time signal acquisition and feature extraction. An alternative strategy employs task-based paradigms or hybrid neuromodulation (e.g., combining TMS with transcranial alternating current stimulation, tACS) to induce predefined neural states that are difficult to capture at rest (Figure 1D). For example, Fehér et al. used tACS phase information to guide TMS timing, mimicking real-time EEG phase control [62]. Gharabaghi et al. triggered TMS pulses using EEG features and tactile feedback, enhancing motor cortex excitability in healthy individuals and stroke patients with mild hand paralysis [63]. EEG signals evoked by closed-loop TMS are termed closed-loop TMS-EEG. By ensuring neural state consistency at stimulation time, this approach improves TMS-EEG reproducibility and captures state-dependent neural responses. Task-embedded TMS-EEG represents a quasi-closed-loop method. By aligning TMS delivery with specific task phases or external stimuli, researchers investigate relationships between neural activity, tasks, and behavior. While not strictly closed-loop, this approach reduces technical complexity and uniquely links brain dynamics to structural/behavioral outcomes.

Previous studies explored brain-behavior relationships using long-term protocols (e.g., rTMS/TBS) to assess how TMS impacts task performance, inferring causal roles of targeted regions [64]. However, disrupting neuronal activity only reveals regional causality, not network interactions. TMS-EEG studies demonstrate that single pulses induce detectable signal propagation across connected regions without behavioral effects [65,66]. Combining tasks with TMS-EEG helps uncover network mechanisms underlying specific cognitive functions [67]. Both closed-loop and task-embedded TMS-EEG belong to the broader category of state-dependent TMS-EEG technologies.

### 3.2. Fundamental Research on Closed-Loop TMS-EEG

Closed-loop TMS research was commonly categorized by its output measures: cortico-spinal responses and cortical responses.

(1) Cortico-spinal responses (MEPs). Early closed-loop TMS studies primarily evaluate stimulation efficacy using MEPs or behavioral outcomes. Comparing MEP differences further validates connectivity variations in corticospinal synchronization across neural states. Iscan et al. demonstrated that pre-stimulus alpha oscillations correlate with inter-subject MEP variability during sTMS of the hand motor cortex: higher alpha amplitude dispersion corresponds to greater MEP variability [68]. Desideri et al. showed that phase-synchronized TMS with sensorimotor mu rhythms enhances motor-evoked responses in hand muscles [69]. Zrenner et al. proposed that mu rhythm phases reflect distinct corticospinal excitability states. By designing phase-locked rTMS protocols (peak vs. trough mu phases), they confirmed that real-time brain states modulate TMS-induced plasticity [70]. Building on this, Zrenner’s team applied mu-alpha phase-synchronized rTMS/TBS to the left dorsolateral prefrontal cortex (DLPFC) in treatment-resistant major depressive disorder (MDD), demonstrating specific neuromodulatory effects [71]. Beyond mu rhythms, Gordon et al. developed a closed-loop protocol triggering TMS at specific theta phases in the dorsomedial prefrontal cortex (DMPFC), validating its reliability [72]. Closed-loop principles also extend to spatial optimization: ervo et al. created an automated system adjusting figure-of-eight coil angles based on MEP feedback, rapidly identifying optimal angles for maximal MEP amplitudes—a novel application of closed-loop TMS [73].
(2) Cortical responses (TMS-EEG). Despite TMS-EEG’s potential as a direct biomarker of TMS-evoked neural activity, hardware limitations and real-time processing challenges have hindered closed-loop TMS-EEG research. Early efforts, such as Fehér et al.’s alpha-rhythm-tACS-guided TMS-EEG, introduced artifacts from external stimulation, distorting recorded signals [74]. Quasi-closed-loop approaches (e.g., aligning TMS with task/external stimuli) simplify technical demands but risk confounding variables from additional neuromodulation. Recent advances in real-time EEG prediction are revitalizing closed-loop TMS-EEG. Momi et al. retrospectively analyzed whether TMS pulses occurred during the positive or negative phase of mu-frequency oscillations and examined the subsequent effects on interhemispheric connectivity [58]. In Table 1, we focused on recent closed-loop TMS-EEG studies over the past three years, revealing a prevalent use of strategies involving phase-controlled TMS output. The general practice of this strategy was to design a flexible TMS-EEG device and develop an effective phase prediction control algorithm to achieve closed-loop control functionality [75]. In the phase-locked loop TMS-EEG experiment, the EEG signals of the subjects were recorded, the phase information of the current neural activity rhythm was extracted, and based on the predetermined target phase value, the output timing of TMS real-time stimulation was guided to achieve phase-locked control. However, the physiological significance of EEG phase information remains unclear, and using alpha phase as a closed-loop control parameter makes it challenging to correlate TMS-EEG results with the physiological significance of the control parameter. More meaningful control strategies (such as microstates [76], Large-Scale Brain States [77,78]) were also gradually being discussed, and as closed-loop technologies were expected to play a significant role in addressing optimization timing issues in TMS-EEG and related mechanistic discussions, an increasing number of relevant studies will be reported in the future.

### 3.3. Fundamental Research on Task-Embedded TMS-EEG

Bortoletto et al. proposed that when TMS targets brain regions within networks highly correlated with cognitive tasks, TMS-EEG can characterize top-down modulation of functional networks by mapping inter-network information flow [83]. Bergmann et al. validated this using concurrent TMS-fMRI to observe local and remote network effects: task-controlled TMS pulses preferentially propagate responses from stimulated targets to tightly connected nodes engaged during task processing, enabling researchers to distinguish task-specific functional connectivity patterns [67]. These high-spatial-resolution findings support the feasibility of task-embedded TMS-EEG. TMS-EEG reveals dynamic functional connectivity shifts driven by brain states and hierarchical organization of stimulation targets. During cognitive tasks, TMS-EEG captures selective activation of transiently engaged cortical networks, with local TMS effects propagating to connected regions in a cognition-dependent manner. Connectivity between stimulated targets and downstream modules associated with specific cognitive functions is enhanced. Morishima et al. exemplify this approach by using TMS as a probe to assess impulse transmission from the prefrontal cortex (PFC) to specialized posterior regions during attention tasks. Participants attended to specific visual stimulus features while TMS-EEG tracked activation spread. Their hypothesis—that stimulating attention-related PFC regions induces anatomically constrained activation spreading to posterior areas—demonstrates how propagation patterns depend on task-specific functional networks [66]. Task-embedded TMS-EEG holds unique advantages over traditional resting-state protocols for functional neural network imaging. While resting-state TMS-EEG can infer information flow directionality from sequential activations [84], such flows are static and non-functional. In contrast, task-embedded TMS-EEG integrates neural signal properties during cognition, linking methodology to behavior to probe functional networks and cognitive mechanisms. TMS transiently modulates task-relevant networks [45,85], while EEG records real-time inter-network interactions. This combination enables precise mapping of information flow across brain regions to identify task-critical connectivity.

Key strengths of task-embedded TMS-EEG include millisecond temporal resolution for direct measurement of electrophysiological signals during cognition; dynamic connectivity insights, revealing state- and target-dependent functional network reconfigurations; applications across experimental contexts: visual processing [86], attention [87], and emotion regulation [88]. We summarized recent studies in Table 2 over the past three years.

However, task-embedded TMS-EEG remains underexplored. A critical limitation is the arbitrary timing of TMS pulses during tasks, which compromises its high temporal resolution potential. Optimizing stimulation timing protocols to align with task phases or neural oscillations will be essential for future advancements.

## 4. State Dependency of TMS-EEG: Conceptual Framework

State-dependent TMS-EEG represents the future direction of this technology, with its core objective being to clarify how TMS-EEG outcomes depend on the neural state at the moment of stimulation. By addressing this dependency, researchers aim to enhance the performance of TMS-EEG as a neuroimaging tool, refining its reliability and applicability in both basic neuroscience and precision medicine. Temporal variability remains a critical barrier to the widespread adoption of TMS-EEG. High-precision TMS protocols are essential to minimize confounding effects from irrelevant neural activity. Previous optimization efforts focused on spatial refinement—improving targeting accuracy through advanced coil designs and neuronavigation systems [32]. However, spatial precision alone is insufficient. Attention is now shifting toward temporal optimization strategies, such as aligning TMS pulses with specific neural states (e.g., oscillatory phases) to reduce variability and enhance reproducibility. Beyond stabilizing neural states, state-dependent TMS-EEG aims to capture transient activations of task-related brain networks with millisecond resolution. By using cognitive tasks to engage specific neural circuits, researchers can probe dynamically activated networks during task execution. However, task-related network activations are often brief, and mistimed TMS pulses may fail to capture these transient states. Improving the temporal precision of task-embedded TMS-EEG—identifying optimal stimulation windows—is crucial for mapping functional networks and understanding their role in cognition.

For this purpose, we have summarized several major scientific issues and challenges of state-dependent TMS-EEG.

### 4.1. Major Scientific Issues in State-Dependent TMS-EEG

**Figure 2:**
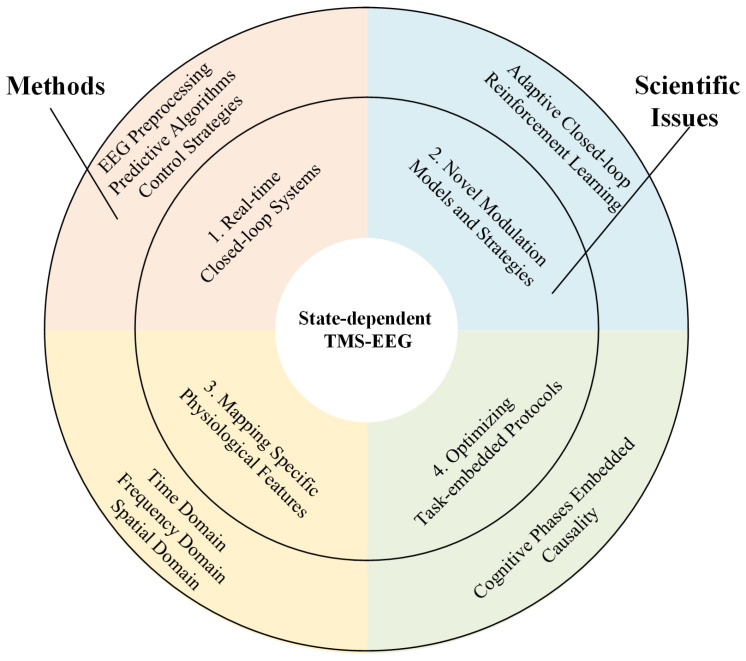
Framework of scientific issues for state-dependent TMS-EEG.

As illustrated in Figure 2, the major scientific issues in state-dependent TMS-EEG comprise four key aspects: optimization of real-time closed-loop systems, designing of novel modulation models and strategies, mapping of specific physiological features, and optimization of task-embedded protocols, each of which will be discussed below.

(1) Designing real-time closed-loop systems that integrate low-latency EEG preprocessing, predictive algorithms for neural state estimation, and appropriate control strategies to synchronize TMS pulses with target brain states. Traditional TMS-EEG techniques cannot precisely synchronize brain activity states at the moment of TMS stimulation, resulting in different initial states of induced TMS-EEG components. In contrast, closed-loop TMS delivers stimuli in sync with brain activity states, yielding closed-loop TMS-EEG data with a locked initial state. Closed-loop TMS-EEG technology comprises three fundamental components: selecting appropriate feedback signals to represent the current neural activity state, establishing precise and effective real-time closed-loop control strategies, and recording observational TMS-EEG signals. Due to the fact that EEG signals collected by the closed-loop TMS-EEG system are only available up to the present moment and there is a delay in signal acquisition and transmission, it is challenging to capture the current neural activity trends and predict future trends. Therefore, a time series forecasting approach is needed to extrapolate the EEG signals. Although EEG signals are non-stationary, the short-term brain information can be considered stationary, making brain signal prediction feasible. A commonly used method involves using an autoregressive model to forecast brain signal sequences, as shown in Formulas (1)–(5).

An autoregressive model is a common method for analyzing time series data, where the sequence is predicted using the sequence itself, known as autoregression. For a stationary process {$x_{1},x_{2},\cdots ,x_{T}$}, it satisfies the following formula, where the random variable at time t can be expressed as a linear combination of the random variables *X_t_* at previous times {t−p,⋯,t−2,t−1}.

(1) $$x_{t}=a_{1}x_{t-1}+a_{2}x_{t-2}+a_{3}x_{t-3}+\ldots a_{p}x_{t-p}+\delta_{t}$$

In the equation, δ represents a white noise sequence, $a_{1},a_{2},\cdots ,a_{T}$ denotes the coefficients of the autoregressive model. The sequence is then structured in matrix form as:

(2) $$X=\left(\begin{array}{ccc} x_{P} & \ldots & x_{1}\\ \vdots & \ddots & \vdots \\ x_{N-1} & \cdots & x_{N-P} \end{array}\right),\;Y=\left(\begin{array}{c} x_{p+1}\\ x_{p+2}\\ \vdots \\ x_{N} \end{array}\right),\;A=\left(\begin{array}{c} a_{1}\\ a_{2}\\ \vdots \\ a_{p} \end{array}\right)$$

Thus, it satisfies:

(3) Y=XA+δ

Satisfying the condition of minimizing residuals in the least squares estimation, setting = 0, the coefficients of the autoregressive model can be expressed as:

(4) $$A={\left(X^{T}X\right)}^{-1}X^{T}Y$$

A sequence {$x_{m+1},x_{m+2},\cdots ,x_{m+T}$} of length *T* after time m can be estimated using the following formula:

(5) $$\left(\begin{array}{c} x_{m+1}\\ x_{m+2}\\ \vdots \\ x_{m+T} \end{array}\right)=\left(\begin{array}{ccc} x_{m-T+1} & \cdots & x_{m-T-p+1}\\ \vdots & \ddots & \vdots \\ x_{m} & \cdots & x_{m-p} \end{array}\right)*A$$

(2) Achieving true closed-loop control requires developing novel modulation models and strategies. As the adaptive closed-loop brain stimulation proposed by Roesch et al., which dynamically adjusts controller parameters in real time based on the evolving relationship between stimulation inputs and system outputs [96]. However, this strategy still differs from strictly defined engineering closed-loop control, as TMS cannot actively drive EEG signals towards desired states in real time. In contrast, Humaidan et al. conducted a proof-of-concept experiment [97], pioneering the integration of closed-loop EEG-TMS with a reinforcement learning (RL) algorithm, specifically Deep-Q Learning. Targeting a two-node brain network (M1 and SMA) to enhance SMA-to-M1 facilitation, the algorithm autonomously optimized stimulation parameters (sensorimotor mu-rhythm phase) using observed facilitation levels as the reward signal. Different agents (simple Q-learning table and deep Q-learning) both successfully learned to identify the optimal stimulation phase, demonstrating RL’s feasibility for goal-directed real-time parameter optimization. The specific steps of the RL algorithm in the original text were as follows:

Environment: The target network consists of the SMA and the M1. Paired-pulse stimulation was applied to evaluate SMA-to-M1 facilitation, a measure of effective connectivity. SMA stimulation was optimized for maximum facilitation, and coil positions were monitored using a neuronavigation system.

Action: The action to optimize was the phase of the sensorimotor mu-rhythm used to trigger rTMS. Eight discrete phase bins were defined for sampling, as different EEG phases correspond to varying excitability states of the brain.

Observation: The primary output was the SMA-to-M1 facilitation ratio, calculated as the ratio of the mean MEP amplitude from paired-pulse SMA-M1 stimulation to the mean MEP amplitude from single-pulse M1 stimulation.

Algorithm: A Deep-Q Learning (DQN) algorithm was used to approximate the query function. The agent learned by interacting with the environment, selecting actions (phase bins) to maximize cumulative rewards based on the observed facilitation.

Reward: At each step, the agent received a reward based on the observed facilitation. The target facilitation was set at 1.5 times the baseline SMA-to-M1 facilitation, and the reward function guided the agent to select the optimal mu-rhythm phase bin for the next rTMS pulse.

**Figure 3:**
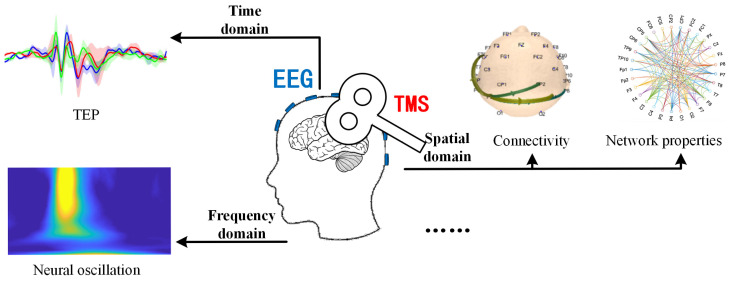
Typical physiological characteristics of TMS-EEG.

(3) Investigating causal mechanisms by combining multiscale EEG analyses with pharmacological or computational approaches to dissect how brain states modulate TMS-evoked responses. Common physiological indicators of TMS-EEG can be categorized into three classes based on time domain, frequency domain, and spatial domain. The representative characteristics of these divisions are illustrated in Figure 3: the most typical features in the time domain include the temporal waveforms of TEP components and their whole-brain topographical distributions; in the frequency domain, typical features include neural oscillations (intrinsic frequencies); and in the spatial domain, it encompasses functional connectivity between different brain regions and network properties of brain functional networks. By mapping specific physiological features of TMS-EEG to actual functional states such as cognitive function, motor function, consciousness state, emotional state, and rehabilitation status, it is possible to construct a neural state-regulation strategy-cognitive function model based on state-dependent TMS.
(4) Optimizing task-embedded protocols to align TMS pulses with distinct cognitive phases (e.g., perception, decision-making), enabling dynamic mapping of functional networks and their behavioral relevance through time-resolved connectivity tools (e.g., time-varying Granger causality [98]). By delivering TMS pulses at different time points during task processing, the originally static functional connectivity features are linked together at different stages of task processing. This dynamic presentation of TMS-EEG network connections over time forms a changing connectome [90]. This innovative technique extends TMS-EEG into the temporal dimension during tasks with the aim of enhancing the temporal resolution that may be compromised in task-related TMS-EEG studies due to the arbitrary timing of TMS pulses.

### 4.2. Challenges

How to design a closed-loop transcranial magnetic stimulation (TMS) system based on real-time EEG, addressing the challenges of complex online EEG physiological feature extraction and real-time TMS output, is the primary issue and technical bottleneck in the development of state-dependent TMS technology. This involves optimizing the time complexity of EEG signal preprocessing algorithms and feature extraction methods, building high-speed parallel computing platforms, selecting accurate EEG time-series prediction methods, and formulating appropriate control strategies. These components require separate investigation, including recording EEG data prior to TMS stimulation using TMS-compatible EEG amplifiers and transmitting it in real time to a high-speed parallel computing platform, rapidly extracting online neural features (such as neural rhythm phases, cortical activation levels like specific rhythm energy, microstates, synchrony indices and functional connectivity [99]), and selecting appropriate control strategies to adjust various TMS output parameters. The optimization design of a closed-loop TMS control platform involves real-time recording of the execution time of closed-loop algorithms and the computational resources invoked by the hardware, enabling adjustments to the high-speed parallel computing platform to improve execution efficiency. At the same time, the closed-loop control algorithm should be optimized based on the control error of the actual TMS output to enhance the accuracy of the closed-loop control. While large computer clusters or high-performance computing servers can efficiently meet the requirements of closed-loop TMS control, their high cost makes widespread implementation impractical. Leveraging GPU (Graphics Processing Unit) acceleration provides a more accessible and cost-effective design solution.

## 5. Conclusions

This review synthesizes the foundational concepts and application paradigms of state-dependent TMS-EEG. Moving forward, a pivotal research focus will be leveraging state-dependent protocols to systematically investigate how neural states at the time of TMS delivery shape evoked EEG responses. Anticipated outcomes include: (1) enhanced stability and reproducibility of TMS-EEG experiments by mitigating variability from spontaneous neural fluctuations; (2) improved dynamic detection capabilities in task-embedded paradigms to track transient network activations during cognitive processing; and (3) exploration of novel temporal stimulation frameworks (e.g., phase-locked or task-phase-aligned protocols) to expand TMS-EEG applications in brain mapping and clinical interventions. Collectively, these advancements will propel the technical evolution and translational potential of TMS-EEG, bridging mechanistic insights into brain dynamics with personalized neuromodulation strategies. If these advancements can be realized, they will bridge the chasm between static neuroimaging and dynamic, state-aware brain mapping, unveiling fresh insights into brain function and personalized therapeutic interventions.

## Figures and Tables

**Table 1:** Recent closed-loop TMS-EEG studies.

Authors	N	Strategy	Main Result(s)
Ding et al. [79]	30	Alpha-phase-locked TMS with O1 electrode	1. Closed-loop TMS can improve the stability between trials, thereby reducing the variability of TMS-EEG data results. 2. Multiple time features, frequency features and spatial features of TMS-EEG were influenced by the alpha phase at the time of stimulation.
Perera et al. [80]	18	Alpha-phase-locked TMS with Hjort-Laplacian C3 electrode	1. MEP amplitude was positively influenced by alpha power and phase, exhibiting more robust responses during the trough phase and high power levels. 2. Alpha power and phase had an impact on TEP characteristics.
Bigoni et al. [81]	20	Mu/Beta-phase-locked TMS with Hjort-Laplacian C3 electrode	1. Pre-TMS mu oscillatory power and phase significantly predicted both early and late cortical EEG responses. 2. Pre-TMS beta power significantly predicted early and late TEP components.
Erickson et al. [82]	19	Alpha-phase-locked TMS with C3 electrode	1. Alpha-phase resetting and N100 amplitude depended on TMS intensity and were significant versus peripheral auditory sham stimulation. 2. Alpha-phase inversion after stimulations near peaks but not troughs in the endogenous rhythm.
Ding et al. [76]	21	Microstate	The N100 component of microstate C group was significantly higher than of microstate D group, and the P180 component of microstate D group was significantly higher than of microstate B groupand slightly higher than of microstate C.
Marzetti et al. [77]	-	Fast-Dynamic Large-Scale Brain States identified by Hidden Markov Model (HMM)	Introduced the Endpoint-Related Fast-Dynamic Large-Scale Brain State (ER-FLBS) concept–a dynamic whole-brain network state that serves as a spatiotemporal reference for optimizing TMS efficacy.
Makkinayeri et al. [78]	20	Fast-Dynamic Large-Scale Brain States identified by HMM	1. A significant link between rapid transient large-scale brain networks and corticospinal excitability. 2. MEPs were enhanced when the motor network was more active pre-stimulation.

**Table 2:** Recent task-embedded TMS-EEG studies.

Authors	N	Strategy	Main Result(s)
Fernandez-Linsenbarth et al. [89]	27 healthy controls and 22 patients	Auditory oddball task	The task-related cortical activity modulation deficit in schizophrenia patients: Schizophrenia patients showed higher cortical reactivity following transcranial magnetic stimulation single pulses over the left dorsolateral prefrontal cortex compared to healthy controls.
Ding et al. [90]	30	Visually guided gap saccade task	Significant differences in information flow in the gamma bands TMS-EEG data at different task stages.
Casula et al. [91]	22	Go/NoGo task	1. A task-specific bidirectional alteration in theta/gamma connectivity between the supplementary motor cortex (SMA) and M1 was observed. 2. When participants were instructed to suppress their responses, distinct N100 complements emerged.
Fong et al. [92]	32	Cerebellar TMS during visuomotor adaptation	The P80 and N110 peaks were consistently observed in the cerebellar motor learning experiment, exhibiting varying amplitudes across different stages of learning.
Bianco et al. [93]	14	Go/No-Go task	When TMS was applied over the SMA during the late Bereitschaftspotential phase, increased cortical activity was observed based on source reconstruction within the stimulated region.
Guidali et al. [94]	25	Action observation task	Under normal conditions, the M1 alpha network recruited during left-hand movement observation is significantly stronger than during static hand observation. Beta connectivity during left-hand movement observation is suppressed compared to all other conditions.
Zazio et al. [95]	20 healthy controls and 20 patients	Visuo-tactile spatial congruency (VTSC)	Subjects presented altered TEP connectivity patterns during both touch perception and touch observation, with no differential modulation between human-directed and object-directed touches in the observation condition.

## Data Availability

No new data were created or analyzed in this study. Data sharing is not applicable to this article.
